# Neoadjuvant docetaxel, cisplatin and ifosfamide (ITP) combination chemotherapy for treating penile squamous cell carcinoma patients with terminal lymph node metastasis

**DOI:** 10.1186/s12885-019-5847-2

**Published:** 2019-06-25

**Authors:** Jian Xu, Gang Li, Shi Miao Zhu, Qi Liang Cai, Zhun Wang, Xiong Yang, Hong Tan Zhang, Yuan Jie Niu

**Affiliations:** 1 Department of Urology, Tianjin Institute of Urology, The second hospital of Tianjin Medical University, Tianjin, 300211 China; 2 Department of Urology, Tumor Hospital of Shandong Province, Jinan, 250117 China

**Keywords:** Penile cancer, Lymph node metastasis, Neoadjuvant chemotherapy, Surgery, Penile squamous cell carcinoma

## Abstract

**Background:**

Chemotherapy may be a valuable treatment option as neoadjuvant treatment for locally advanced penile cancer according to some previous studies, but the rarity of the sample and the Lack of large-scale clinical trials hampered the attempt to establish a solid evidence base for its routine use. The purpose of this study was to evaluate the efficacy of the neoadjuvant chemotherapy combined with a ITP regimen including docetaxel, cisplatin and ifosfamide for treating advanced penile cancer patients.

**Methods:**

A total of 19 patients who were classified into advanced penile cancer (PN3) received neoadjuvant chemotherapy of ITP regimen from June 2009 to June 2016 in our hospital.

**Results:**

After chemotherapy 12 patients had a partial response (PR), 5 had stable disease (SD) and progressive disease (PD) in 2 cases. The 12 responders underwent penectomy, bilateral inguinal lymphadenectomy (ILND) and pelvic lymph node dissection (LPLND). In contrast, 7 cases who were non-responsive received palliative local radiotherapy. After a median follow-up of 30.6 months, there was statistically significant improvement in median PFS and OS among patients who experienced an objective response to neoadjuvant chemotherapy (group A) compared with those patients who did not respond to chemotherapy (group B) (log-rank test; *P* < 0.001).

**Conclusion:**

Neoadjuvant docetaxel, cisplatin and ifosfamide chemotherapy gave 63% (12/19) of patients who were diagnosed with stage n3 penile cancer the chance of radical resection of metastases, and their OS and PFS were significantly higher than those who could not be operated on and the therapeutic dose, toxic and side effects are acceptable in the Chinese Han population. Therefore, neoadjuvant ITP chemotherapy in the treatment of stage T3 penile cancer patients may have cheerful prospects in the Chinese Han population.

## Background

Squamous cell carcinoma (SCC) of the penis is a relatively rare and aggressive disease in the developed countries, but higher incidences have been observed in the developing countries [1, 2]. Perhaps due to benign initial symptoms, embarrassment and insufficient awareness or knowledge, a considerable proportion of patients with penile cancer had a patients’ delay of more than 6 months, which lead 25% of men to present with advanced disease at the time of diagnosis. The lymph node metastasis of penile cancer occurs first in the groin lymph nodes. The greatest prognostic factors in SCC of penis are the presence and extent of lymph node involvement [3]. Whether exist the regional lymph node metastasis, the extent of metastasis and whether can undergo radical resection are the determinant of the survival rate. For patients with fixed inguinal lymph node (LN) metastasis or pelvic lymph node (N3) involvement, upfront operation is not recommended because of the small possibility of cure, short survival time and wide range of operation. Upfront chemotherapy seems to be a more reasonable method [3]. The aim of neoadjuvant chemotherapy is to reduce the size and infiltration of tumors, to improve the effect of resection, to alleviate surrounding injury and to eliminate distant micro-metastasis. According to previous studies, chemotherapy as a neoadjuvant therapy for locally advanced penile cancer may show good results, but the scarcity of samples and the lack of large-scale clinical trials hinder attempt to establish a solid evidence base for its routine use [4] . This should strengthen research and further improve the related chemotherapy regimen.

Neoadjuvant chemotherapy is now commonly used in penile cancer with locally invasive or fixed inguinal lymph nodes as well as in treatment and palliation for advanced or metastatic disease [5]. In the neoadjuvant treatment, up to 69% of the patients who were clinically unresected had an objective response allowing them to be clinically removed [6]. Multiple regimens had been studied and implemented. The BMP regimen (bleomycin, methotrexate and cisplatin) being most commonly used had a 33–63% response rate which had definite therapeutic effect, but a confirmatory study reported that the treatment might be highly toxic and modest effect [7]. In head and neck cancer, neoadjuvant combination regimens containing taxanes produced high-response rates and improved survival outcomes in patients with unresectable disease [8, 9]. The chemotherapy regimens containing taxanes also Showed promising results to Penile cancer. The TPF protocol, including cisplatin (DDP), 5-fluorouracil and paclitaxel, is becoming widely accepted as the first-line treatment option because of good efficacy and low toxicity. However, a new study suggested that neoadjuvant TPF-chemotherapy obtained an imaging-based response in 60% of patients. However, pathologic complete response rate was only 4%. The 2-year PFS and DSS probability were only 12 and 28%, respectively. Toxicity was considerable in every study patient. TPF chemotherapy should be used with caution because of poor tolerance and disappointing survival rates [10]. Bermejo and his colleagues reported that four-fifths of patients receiving ITP neoadjuvant therapy (paclitaxel, cisplatin and ifosfamide) had a complete response, and three of them had a histologically confirmed complete response [11]. A larger sample study in the United States showed that 30 men received ITP treatment, of which 15 (50.0%) had an objective response, 22 (73.3%) underwent surgery and 9 had long-term survival [12].

To further evaluate the efficacy of neoadjuvant chemotherapy for penile cancer, we conducted a retrospective study of a combination of ITP administered before surgery for fixed inguinal lymph node metastasis or involved pelvic LN(N3) in patients with SCC of penis in China.

## Methods

### Patients

The vast majority of penile cancer is squamous cell carcinoma and other uncommon malignant penile diseases include Paget disease, basal cell carcinoma and melanoma. Following institutional review board approval, 19 patients who were recruited in our study were squamous cell carcinoma of the penis, which were confirmed by fine needle aspiration biopsy and treated from June 2009 to June 2016 in a single medical institution comprised the initial study cohort. The patients were classified into the stage PN3 SSC of the penis according to the 2009 TNM classification of malignant tumors (UICC International Union Against Cancer seventh edition). The pathological grade for SCC of penis was defined as well differentiated, moderately differentiated or poorly differentiated in accordance with Broders’classifcation standards [13].

The recruited patients aged 35–69 years and with histologically proven SCC of the penis were clinically staged TxN3M0 according to 2009 TNM classification. All of the patients in this study had fixed inguinal lymph node metastasis which specific manifestations included palpation of inguinal region with fixed masses, CT or PET-CT showed enlarged and fused lymph nodes which had rough and irregular margins and invaded the surrounding femoral vessels and Inguinal skin. In addition to having a fixed inguinal lymph node, two of the 19 patients had pelvic lymph node metastasis before treatment (the basic and initial characteristics of the patients who were studied are shown on Table 1). Other eligibility criteria included: the penile local carcinoma had been or was able to be removed surgically; Patients have not received previous radiation therapy or systemic chemotherapy for penile carcinoma containing cisplatin or paclitaxel; Physical and functional assessments of the patients could withstand surgery and chemotherapy.

**Table 1:** Baseline characteristics of patients (*N* = 19)

Characteristics	amount	Percentage(%)
Age (years)		
Median	56.1	
Range	35–69	
Course of disease		
Average (months)	6.6	
Range	40 days-2 years	
UICC staging		
T1	4	21.1
T2	7	36.8
T3	7	36.8
T4	1	5.3
Lymph node stations clinically involved		
Fixed inguinal lymph node	17	89.5
Fixed inguinal lymph node + pelvic lymph node enlargement	2	10.5
Broders’classifcation		
Well differentiated	4	21.1
Moderately differentiated	7	36.8
Poorly differentiated	8	42.1
Clinical response to chemotherapy		
Progression	2	10.5
Stable disease	5	26.3
Partial or complete response	12	63.2
Treatment after neoadjuvant chemotherapy		
ILND+PLND	5	26.3
Penile partial resection + ILND + PLND	6	31.6
Penectomy + ILND + PLND	1	5.3
Radiotherapy	7	36.8
Recurrence and metastasis after treatment		
pelvic lymph node metastasis only	2	10.5
pelvic lymph node + bone metastasis	2	10.5
pelvic lymph node + pulmonary metastasis	3	15.8
pelvic lymph node + liver metastasis	3	15.8
widespread lymph node metastases	1	5.3

### Chemotherapy

Since June 2009, Neoadjuvant chemotherapy of the ITP regimen was provided for the selected patients with a written informed consent. The regimen consisted of up to 2–4 cycles every 21 days with docetaxel 75 mg/m^2^ on day 1, cisplatin 25 mg/m^2^ on days 1 to 3 and ifosfamide 1200 mg/m^2^ on days 1 to 3.

After 2 cycles of chemotherapy, the clinical tumor response was evaluated with contrast-enhanced computed tomography (CT) and assessed using the response evaluation criteria in solid tumors (RCIST), version 1.1 [14]. The objective tumor response to neoadjuvant chemotherapy was defined as decrease (PR) or disappearance (CR) of the sum of diameters of metastatic lymph nodes, or metastatic lymph nodes became mobile after chemotherapy through physical examination and CT. Non-responsive to neoadjuvant chemotherapy was defined as metastatic lymph nodes continued to enlarge (PD) or be stable (SD).

The adverse events associated with chemotherapy and surgery were graded according to the National Cancer Institute Common Terminology Criteria for Adverse Events version 4.0. Regular blood tests, including renal function, white blood cell and platelet counts, were done to monitor toxicity.

### Subsequent treatment

Partial or total penectomy, inguinal and pelvic lymphadenectomy were performed in patients who were responsive to neoadjuvant chemotherapy in an attempt to remove all residual malignant tissues. Postoperative patients received adjuvant chemotherapy with the same regimen of preoperative neoadjuvant chemotherapy that included 2–4 cycles. Either volumetric modulated arc therapy (VMAT) or intensity modulated radiotherapy (IMRT) (dose of 55–70 Gy, 1.8 Gy daily) was performed for the cases that were non-responsive after chemotherapy.

### Clinical end points

The main end points of the study were progression-free survival (PFS) and overall survival (OS). PFS was defined as time from beginning of chemotherapy until clinically or radiologically documented disease progression or death from any cause. Patients alive without progression at last date of follow-up were censored. OS was defined as the period of time from the start of chemotherapy to patient death of any cause.

PFS and OS were calculated with Kaplan–Meier survival curves. The log-rank test was used for analysis of the differences of survival between the responsive group (Group A) and the non-responsive group (Group B). All analyses were performed using SPSS (version 22.0; SPSS Inc., Chicago, IL). *P* < 0.05 was considered statistically different.

## Results

### Patient characteristics

During June 2009 to June 2016, a total of 19 patients were received neoadjuvant ITP chemotherapy. All had histologically proven squamous cell carcinoma. 5 patients had previously received partial amputation of the penis at other medical institutions, whose fixed inguinal lymph node metastases were found later. The remaining 14 penile cancer patients had the penile local carcinoma and fixed inguinal lymph node metastasis when admitted to our hospital. All patients underwent full-body CT or PET-CT examinations before treatment, and two of them were found to have pelvic lymph node metastases. No distant metastases were found. Characteristics are reported in Table 1.

### Therapy

Four patients completed four cycles of ITP. After two cycles of chemotherapy, the four patient’s condition was stable, and then two cycles of chemotherapy were carried out. Three patients completed only one cycle because of disease progression (*n* = 2) and toxicity (*n* = 1). Twelve patients completed two cycles and because of the good therapeutic effect, operations were carried out subsequently. Of the evaluable patients, twelve of the 19 patients achieved PR, 5 achieved SD and 2 had progressive disease after chemotherapy. Among the 12 responders who received surgery, five cases underwent bilateral inguinal lymphadenectomy (ILND) and pelvic lymph node dissection (PLND), six cases received partial amputation of the penis and ILND+PLND, and one case underwent penectomy and ILND+PLND (Table 1).

### Side effects

The patient’s overall tolerance to chemotherapy was good. There were no deaths related to the treatment protocol. There was only one patient who had discontinued chemotherapy due to severe myelosuppression, followed by surgical treatment after one cycle chemotherapy. The major complications following surgery were lower extremity lymphedema. The specific performance was shown in Table 2.

**Table 2:** Adverse effects in patients following chemotherapy and surgery

Adverse effects	Grade 1, *n* (%)	Grade 2, *n* (%)	Grade 3, *n* (%)	Grade 4, *n* (%)
Toxicity of chemotherapy				
Myelosuppression	7 (19)	7 (19)	2 (19)	1 (19)
Nausea/vomiting	10 (19)	5 (19)	1 (19)	0 (19)
Allergic reaction	0 (19)	1 (19)	0 (19)	0 (19)
Myocardial ischemia	1 (19)	1 (19)	0 (19)	0 (19)
Alopecia	2 (19)	2 (19)	0 (19)	0 (19)
Motor neuropathy,	1 (19)	1 (19)	0 (19)	0 (19)
Complications related to operation				
Lower Extremity Edema	6 (12)	4 (12)	0 (12)	0 (12)
Edema in Trunk and/or Genitalia	3 (12)	1 (12)	0 (12)	0 (12)
Delayed wound healing	4 (12)	3 (12)	0 (12)	0 (12)
Lymphocyst	1 (12)	0 (12)	0 (12)	0 (12)

### Posttreatment conditions and follow-up

Postoperative pathology showed pelvic lymph node metastasis in 3 cases, in which including preoperative 2 cases. All 19 cases were followed-up for 11–79 months (average 39.6 months). During this period, 11 cases had pelvic lymph node recurrence or metastases, and then had both regional and systemic metastases (Table 1).

**Fig. 1:**
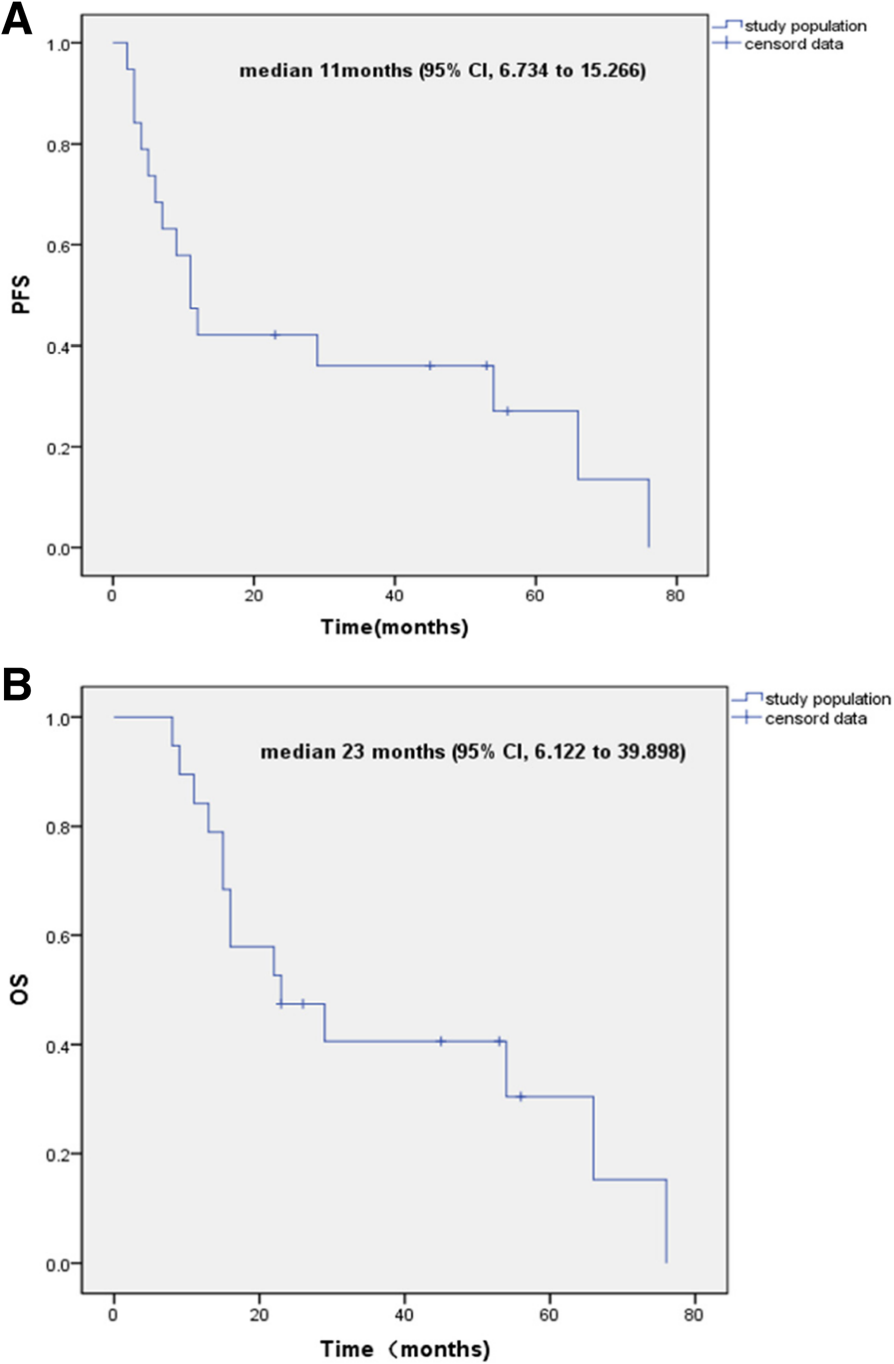
Kaplan-Meier curves show PFS and OS in all patients

**Fig. 2:**
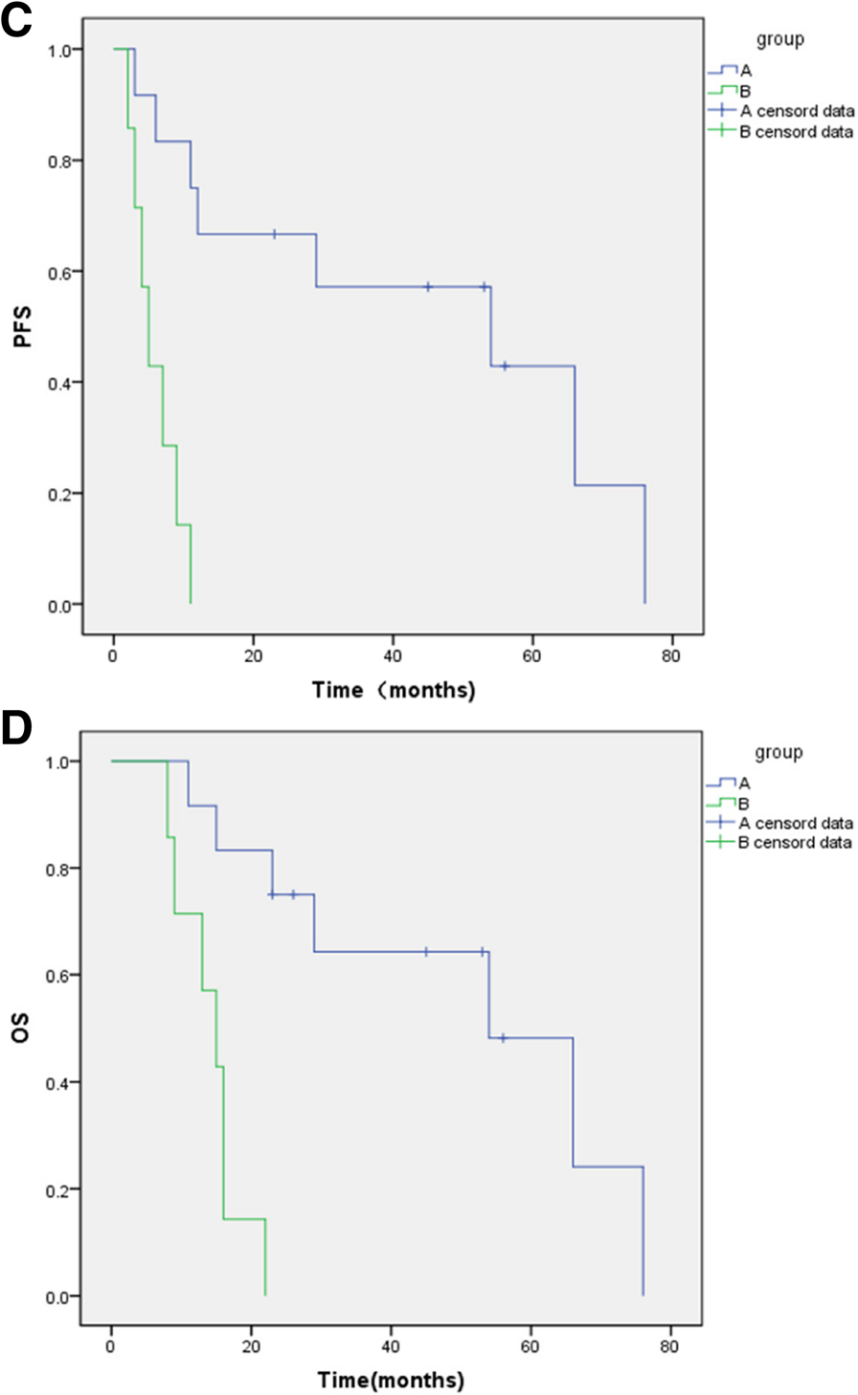
Kaplan-Meier curves show PFS and OS in patients who experienced an objective response (group A) to neoadjuvant chemotherapy compared with those among patients who did not (group B)

After a median follow-up of 39.6 months, 11 of the 19 patients have progressed, and 14 patients have died, with an estimated median PFS of 11 months (95% CI, 6.734 to 15.266; Fig. 1a) and OS of 23 months (95% CI, 6.122 to 39.898; Fig. 1b),respectively. There was statistically significant improvement in median PFS and OS among patients who experienced an objective response (group A) to neoadjuvant chemotherapy compared with those among patients who did not (group B)(log-rank test; *P* < 0.001)(Fig. 2c and d).

## Discussion

As previously mentioned, a lot of penile cancer patients had lymph node metastasis when newly diagnosed. The natural course of penile cancer is a progressive violation of the penis and then spread through the lymphatic vessels to the inguinal lymph nodes, ultimately involving the pelvic lymph nodes. The study of penile lymphangiography showed that the lymphatic metastasis of the penile carcinoma was from the inguinal superficial to deep inguinal lymph nodes and finally transferred to the pelvic lymph nodes, but did not find lymphatic jump transfer [15]. If untreated, the lymph node of the inguinal region will further expand and form an ulcer or tumor on the local skin that will invade the adjacent blood vessels and lead to blood and distant metastasis [16]. According to previous studies, whether the transfer of lymph nodes, degree of metastasis and whether the radical resection are the most important factors in determining prognosis [17, 18]. The occurrence of lymph node metastasis of penile cancer patients with the number of positive lymph nodes, unilateral or bilateral inguinal lymph node metastasis and whether there are pelvic lymph node involvement and lymph node invasion determine a different prognosis [19]. Pandey et al. [20] analyzed 102 cases of lymph node metastasis of penile cancer patients, which the results showed that 1 to 3, 4 to 5, 5 or more lymph node metastasis 5-year survival rates were 75. 6%, 8. 4%, 0, respectively. Studies have also shown that patients with inguinal lymph node metastasis had the 5-year survival rate of < 60% and the 5-year survival rate in pelvic lymph node metastasis was < 30% [21]. Therefore, early detection and treatment can lead to better prognosis. But just as previously described, due to benign initial symptoms, shame and insufficient awareness, a lot of the patients postponed treatment. In this study, the average course of disease was 6.6 months (range 40 days–2 years).

Lymph node metastasis is an important prognostic factor in patients with penile cancer, so the treatment of lymph nodes is essential. Fraley et al. [22] found that lymph node-positive patients with lymph node dissection in a 5-year disease-free survival rate of 75%, while the extension of surgery was 8%. Therefore, patients with pathologically confirmed lymph node metastasis, if safely and completely removed, should be immediately treated lymph node dissection to improve the prognosis of patients. Ravi et al. [23] found that up to 57% of patients with more than 3 positive inguinal lymph nodes had pelvic lymph node metastases. Therefore, the patients with ≥2 positive inguinal lymph nodes were recommended to be added pelvic lymph node dissection [3]. The patients with fixed inguinal lymph nodes or limited pelvic lymph node metastasis (N3) were recommended radiotherapy and chemotherapy combined surgery to achieve the basic principle which is to completely remove the lesion as much as possible. All of the studied patients were diagnosed with stage PN3 SSC of the penis. The responsive group (12 cases) were undergone Partial or total penectomy +lymph node dissection + pelvic lymph node dissection after the neoadjuvant chemotherapy, and the incisal margins were negative, which were in line with the principle of treatment. Postoperative pathology showed pelvic lymph node metastasis in 3 cases and the 3 cases all had pelvic lymph node recurrence or metastases later. Followed-up for average 39.6 months, four cases had pelvic lymph node recurrence or metastases in responsive group and then had both regional and systemic metastases, there was only one survivor (26 months) so far. Therefore, whether is meaningful for the pelvic lymph node-positive patients with pelvic lymph node dissection need further study.

Systemic chemotherapy can be used for lymph node metastasis of neoadjuvant therapy and adjuvant therapy. While the former can achieve tumor shrinkage small to achieve the purpose of complete resection of the operation, the latter mainly used for the multiple lymph node metastasis after surgery [24]. As the low incidence of penile cancer there was no large-scale literature published to guide the treatment of chemotherapy. Therefore, there is no standard treatment program so far. Through a variety of chemotherapeutic drugs in the treatment of penile cancer research, cisplatin-based combination of neoadjuvant chemotherapy can control the disease, reduce the lesion and improve the surgical results. In 1991, Dexeus and colleagues [25] reported that cisplatin (20 mg/m2 day 2–6) ,methotrexate (200 mg/m2 day 1 and 15) and bleomycin (10 mg/m2 day 2–6)(BMP) regimen for advanced squamous cell carcinoma achieved a response in 10 of 14 enrolled patients,which adverse effects were moderate. The regimen was soon widely used for penile cancer. But a series of subsequent studies have found that the program had limited efficacy, high toxicity and high treatment-related deaths [7, 26, 27]. Other regimens were tried later. As previously stated, TPF regimen, including cisplatin, 5-fluorouracil and paclitaxel, also had problems as similar as BMP regimen. Recently, the ITP regimen had been reported. As previously described, in a phase II trial involving 30 pN2/pN3 penile cancer patients treated in the neoadjuvant setting, there were able to achieve an objective response in 50% of men using neoadjuvant ITP (docetaxel, cisplatin and ifosfamide), which was accompanied by acceptable side effects. Ten patients (33.03%) were surviving after a median follow-up of 34 months [12]. On the basis of previous cases [12], a follow up study was implemented by Dickstein and colleagues [17] which broaden additional 31 patients and 5 years of follow-up. All patients were clinical or pathological stage T_any_ and N1–3 penile cancer. Fifty-four patients (88.5%) received ITP chemotherapy and seven received other initial therapy. CRs or PRs were found in 64% of regionally advanced penile cancer patients receiving ITP chemotherapy. OS of patients with objective response to chemotherapy (CR or PR) was 50.1% in 5 years. Despite the optimistic results, there are still many difficulties to overcome in these studies. Despite the optimistic results, there were still many difficulties that need to be overcome in these studies. As the paper said, because of the clinical or pathological stage N1–3, chemotherapy may delay the definitive operation, rendering some unresponsive patients unable to operate. The lack of chemotherapeutic regimen standardization and dose optimization limited the broader application of the ITP regimen. In our study, the recruited patients with histologically proven SCC of the penis were clinically staged TxN3M0, which would have been difficult to resect inguinal and pelvic lymph nodes without objective response for chemotherapy, so that it avoided the possibility that chemotherapy delayed definitive surgery.

Docetaxel and paclitaxel are taxanes. Docetaxel as a new generation of taxane drugs, is a semi-synthetic taxane. Its ability to bind to microtubule proteins is twice that of paclitaxel, which prevents the formation of spindles when cells are mitotic [28]. The accumulation of docetaxel in tumor cells is higher than that of paclitaxel, and the residence time is longer, which is more effective in anti-tumor effect [29]. In vitro tests, docetaxel’s tumor cell reduction rate was three times that of paclitaxel [30]. In the toxic and side effects, docetaxel’s allergic reactions, neurotoxicity and blood toxicity, especially leukopenia are significantly lighter than paclitaxel [31]. In our study, docetaxel replaced paclitaxel. The preoperative regimen consisted of up to 2–4 cycles every 21 days with docetaxel 75 mg/m^2^ on day 1, ifosfamide 1200 mg/m^2^ on days 1 to 3 and cisplatin 25 mg/m^2^ on days 1 to 3. Postoperative patients received adjuvant chemotherapy with the same regimen of preoperative neoadjuvant chemotherapy that included 2–4 cycles. Usually each patient received a total of 6 cycles of chemotherapy, including preoperative and postoperative. Toxicity and side effects were controllable. No deaths which related to the treatment occurred.

In the present study, the overall objective response rate of neoadjuvant chemotherapy was 63% (12/19), similar to that described earlier [17]. Although making use of radiotherapy for non-responsive group, the treatment effect was still poor. The responsive group to chemotherapy had the median OS of 54 months (95% CI 22.035–85.965), while those with non-response had 15 months (95% CI 9.868–20.132). The data for PFS were similar when comparing responders with non-responders. The difference was statistically significant (log-rank test; *P* < 0.001). The survival number of patients in the responsive group was significantly higher than that of non-responsive group, suggesting that neoadjuvant chemotherapy of ITP in combination with surgery have good therapeutic effects for penile cancer patients with advanced lymph node metastasis.

## Conclusions

In conclusion, according to our preliminary study, neoadjuvant docetaxel, cisplatin and ifosfamide chemotherapy gave 63% (12/19) of patients who were diagnosed with stage n3 penile cancer the chance of radical resection of metastases, and their OS and PFS were significantly higher than those who could not be operated on and the therapeutic dose, toxic and side effects are acceptable in the Chinese Han population. Therefore, neoadjuvant ITP chemotherapy in the treatment of stage T3 penile cancer patients may have cheerful prospects in the Chinese Han population. Due to limitations of small sample size further evaluation is necessary. The next study needs to expand the number of specimens and design prospective studies to improve the current research results.

## Data Availability

The datasets are available from the corresponding authors on reasonable request.

## Abbreviations

CR: Complete response
CT: Computed tomography
DSS: Disease-special survival
IMRT: Intensity modulated radiotherapy
OS: Overall survival
PD: Progressive disease
PET-CT: Positron emission tomography- computed tomography
PFS: Progression-free survival
PR: Partial response
SD: Stable disease
VMAT: Volumetric modulated arc therapy
